# Cytotoxicity of the Sesquiterpene Lactones Neoambrosin and Damsin from *Ambrosia maritima* Against Multidrug-Resistant Cancer Cells

**DOI:** 10.3389/fphar.2015.00267

**Published:** 2015-11-09

**Authors:** Mohamed Saeed, Stefan Jacob, Louis P. Sandjo, Yoshikazu Sugimoto, Hassan E. Khalid, Till Opatz, Eckhard Thines, Thomas Efferth

**Affiliations:** ^1^Department of Pharmaceutical Biology, Institute of Pharmacy and Biochemistry, Johannes Gutenberg University of MainzMainz, Germany; ^2^Institut für Biotechnologie und Wirkstoff-ForschungKaiserslautern, Germany; ^3^Department of Pharmaceutical Sciences, Centro de Ciências da Saúde, Universidade Federal de Santa CatarinaFlorianópolis, Brazil; ^4^Institute of Organic Chemistry, Johannes Gutenberg University of MainzMainz, Germany; ^5^Division of Chemotherapy, Faculty of Pharmacy, Keio UniversityTokyo, Japan; ^6^Department of Pharmacognosy, University of KhartoumKhartoum, Sudan; ^7^Institute of Biotechnology and Drug Research, Johannes Gutenberg University of MainzMainz, Germany

**Keywords:** ABC-transporters, Asteraceae, EGFR, p53, Src inhibition, targeted chemotherapy

## Abstract

Multidrug resistance is a prevailing phenomenon leading to chemotherapy treatment failure in cancer patients. In the current study two known cytotoxic pseudoguaianolide sesquiterpene lactones; neoambrosin (**1**) and damsin (**2**) that circumvent MDR were identified. The two cytotoxic compounds were isolated using column chromatography, characterized using 1D and 2D NMR, MS, and compared with literature values. The isolated compounds were investigated for their cytotoxic potential using resazurin assays and thereafter confirmed with immunoblotting and *in silico* studies. MDR cells overexpressing ABC transporters (*P*-glycoprotein, BCRP, ABCB5) did not confer cross-resistance toward (**1**) and (**2**), indicating that these compounds are not appropriate substrates for any of the three ABC transporters analyzed. Resistance mechanisms investigated also included; the loss of the functions of the TP53 and the mutated EGFR. The HCT116 p53^-/-^ cells were sensitive to **1** but resistant to **2**. It was interesting to note that resistant cells transfected with oncogenic ΔEGFR exhibited hypersensitivity CS toward (**1**) and (**2**) (degrees of resistances were 0.18 and 0.15 for (**1**) and (**2**), respectively). Immunoblotting and *in silico* analyses revealed that **1** and **2** silenced c-Src kinase activity. It was hypothesized that inhibition of c-Src kinase activity may explain CS in EGFR-transfected cells. In conclusion, the significant cytotoxicity of **1** and **2** against different drug-resistant tumor cell lines indicate that they may be promising candidates to treat refractory tumors.

## Introduction

Chemotherapy still represents a major treatment option for cancer, despite its limitations in specificity and toxicity. Resistance is an everlasting obstacle in cancer therapy eventually leading to MDR that result to therapy failure. Various factors account for inherent (primary) and acquired (secondary) resistance including: point mutations in target and effector genes, genetic, and epigenetic heterogeneity of cancer cells, increased activity of drug eﬄux pump such as ABC transporters, activation of EGFR leading to increased proliferation by auto-activation of downstream signaling cascades, activation of drug metabolizing enzymes such as CYP450, impairment of DNA repair mechanisms and suppression of apoptosis by mutated p53 (Gottesman, 2002; Holohan et al., 2013).

Resistance to chemotherapy remains a key challenge; therefore, targeted chemotherapy emerged as new concept in the treatment regime. In this treatment, a drug specifically inhibit tumor growth by targeting cellular molecules that are pivotal for growth, progression, and metastasis, for example using monoclonal antibodies or natural or synthetic small molecule inhibitors. This approach increased the specificity of treatment for different types of malignancies, including breast, colorectal, lung, and pancreatic cancers, as well as lymphoma, leukemia, and multiple myeloma (Gerber, 2008).

Natural products considerably impact drug discovery due to their wide structural diversity. More than 60% of clinically used anticancer agents are derived from natural sources (plants, marine organisms, and microorganisms; Newman and Cragg, 2012).

*Ambrosia maritima* L. (*Asteraceae*) is gray-hairy herb, richly branched with finely dissected, fragrant leaves. This plant is widely distributed in the central and northern Sudan, near the banks of Nile River and locally known as *Damsisa.* It is used in folk medicine to treat diabetes, hypertension, urinary tract infections, gastrointestinal disturbances, kidney stones, and also cancer (Khalid et al., 2012; Dirar et al., 2014). *A. maritima* has been planted in Egypt, on canals and banks of the Nile Delta as a molluscocide against the schistosomiasis intermediate host (El-Sawy et al., 1981). Recently, it has been reported that *A. maritima* exhibited considerable cytotoxic activity against various MDR tumor cells (Saeed et al., 2015). Several genera of *Asteraceae* are rich of sesquiterpene lactones (SLs), which are considered as promising class of compounds because of their diverse biological activity with interesting therapeutic mechanisms, including anti-inflammatory, antitumor, antimicrobial, and antiviral properties (Zhang et al., 2005; Fraga, 2011; Merfort, 2011).

In the present work, two pseudoguaianolide SLs, neoambrosin (**1**) and damsin (**2**), were characterized from *A. maritima* L. Their anticancer activities against drug-resistant tumor cell lines were determined, in which either MDR-conferring ABC-transporters [*P*-glycoprotein (*P*-gp), BCRP, ABCB5] or mutation-activated EGFR were overexpressed. Furthermore, drug-resistant TP53-knockout cells were analyzed. The focus of this study was on EGFR-downstream signals due to the striking hypersensitivity CS of EGFR-transfected cells toward **1** and **2**. The two compounds which are structurally related, specifically inhibited c-Src kinase activity, indicating that this oncogenic kinase is a suitable target of **1** and **2**, explaining the CS exhibited by EGFR-transfected cells to these compounds.

## Materials and Methods

### General Experimental Procedures

Pure DCM was used to prepare crude extract from whole plant of *A. maritima.* Crude extract was analyzed by high pressure liquid chromatography (HPLC, Agilent 1100 Series, equipped with a LiChrospher RP 18 column (3 mm × 125 mm; 5 μm, Merck, Darmstadt, Germany) at 40°C and a flow rate of 1 mL/min with an elution gradient composed of H_2_O + 0.1% (v/v) trifluoroacetic acid and acetonitrile. The molecular weight of the selected peaks was determined by HPLC-MS (Agilent 1260 Series LC and 6130 Series Quadrupole MS System). The mass spectra were recorded using atmospheric pressure chemical ionization (APCI) with positive and negative polarization. A Superspher RP 18 (125 mm × 2 mm; 4 μm, Merck) column was used at 40°C. For every run, 1 μL of a sample (1 mg/mL) was injected. Chromabond column (C18 ec-column, Macherey–Nagel, Düren, Germany) was used to obtain the pure compounds. 1D and 2D NMR data were recorded with a Bruker AVANCE III-600 MHz spectrometer equipped with a 5 mm inverse TCI cryoprobe using standard pulse sequences to elucidate the structures of neoambrosin (**1**) and damsin (**2**).

### Plant Material

The whole plant of *A. maritima* was identified and authenticated by Prof. Hassan Khalid at medicinal and aromatic plants research institute (MAPRI) in Khartoum. A voucher specimen (A23) represents the plant was deposited in the herbarium of National Research Institute, Khartoum, Sudan (Saeed et al., 2015).

### Extraction and Isolation of Neoambrosin (**1**) and Damsin (**2**) from the Whole Plant of *A. maritima*

Plant materials (∼100 g) was dried and ground before being subjected to cold maceration with DCM. The plant powder was macerated with overnight gentle shaking in DCM into stoppered flask at room temperature. The extracted solvent was separated and filtered through Whatman no. 1 filter paper. The DCM was evaporated under reduced pressure using rotatory evaporator, resulting in *A. maritima* crude extract.

One gram of the whole plant of *A. maritima* extract was subjected to separation using a silica gel-60 matrix eluting with cyclohexane (C_6_H_10_): Ethyl acetate (EtOAc) gradient in increasing polarities. This resulted into 10 fractions each including: 1 (2 mg), 2 (103 mg), 3 (66 mg), 4 (107 mg), 5 (67 mg), 6 (71 mg), 7 (37 mg), 8 (45 mg), 9 (37 mg), 10 (415 mg). The 10 samples were fractionated via HPLC in micro-titer plates in order to determine their cytotoxic potential. The samples were fractionated using the analytical HPLC method given above. A subsequent located fraction collector (Agilent 1100 Series) was used equipped with 96-well plates for collecting the flow through resulting in 92 different fractions of 250 μl each. These fractions could be used in biological assays. Fractions 3 and 4 of the main column revealed interesting cytotoxic activities (IC_50_ values of 4.5 and 4.8 μM for fractions 3 and 4) against CCRF-CEM cells, respectively. These fractions were subsequently subjected to solid phase extraction using C18- Chromabond column as the stationary phase eluting with neat methanol (MeOH). The resultant fractions were further purified by preparative HPLC using 65% acetonitrile with 35% H_2_O + 0.1% (v/v) trifluoracetic acid for fraction 3, and 60% acetonitrile with 40% H_2_O + 0.1% (v/v) trifluoracetic acid, with 17 mL/min isocratic flow-rate, yielding compounds 1 (20.0 mg) and 2 (50.5 mg), respectively.

### Cell Lines

Drug sensitive CCRF-CEM and multidrug-resistant *P*-gp-overexpressing CEM/ADR5000 leukemic cells were cultured in RPMI-1640 medium supplemented with 10% FBS and 1% penicillin/streptomycin (Invitrogen, Darmstadt, Germany). Doxorubicin (5000 ng/mL) was added to maintain overexpression of *P*-gp (*MDR1*, *ABCB1*) in resistant cells (Kimmig et al., 1990). Breast cancer cells transfected with a control vector (MDA-MB-231-pcDNA3) or with cDNA for the breast cancer resistance protein BCRP/ABCG2 (MDA-MB-231-BCRP clone 23) were cultured and maintained as reported (Doyle et al., 1998). Expression of *ABCG2* in resistant cells was maintained by geneticin (800 ng/mL) (Efferth et al., 2003). Human HEK293-ABCB5 embryonic kidney cells transfected with another ABC-transporter, ABCB5, were propagated in DMEM medium supplemented with 10% FBS and 1% penicillin/streptomycin (Invitrogen) (Kawanobe et al., 2012). Non-transfected HEK293 cells served as control. Human wild-type HCT116 colon cancer cells (p53^+/+^) and knockout clones (p53^-/-^) derived by homologous recombination (Waldman et al., 1995; Bunz et al., 1998) were generously provided by Dr. B. Vogelstein and H. Hermeking (Howard Hughes Medical Institute, Baltimore, MD, USA). Both colon cancer cells were cultured in DMEM medium supplemented with 10% FBS and 1% penicillin/streptomycin (Invitrogen). Wild-type human GBM U87MG cells and cells transfected with control mock vector or an expression vector harboring *EGFR* cDNA with a deletion in exons 2–7 (U87MGΔEGFR), were kindly provided by Dr. W. K. Cavenee (Ludwig Institute for Cancer Research, San Diego, CA, USA; Huang et al., 1997). Six non-transfected human GBM cell lines were used, which overexpress c-Src including: A172, T98G, and U251MG cells, obtained from the German Cancer Research Center (DKFZ, Heidelberg, Germany). DK-MG and SNB-19 cells were kindly provided by Dr. Tcholpon Djuzenova (Department of Radiation Oncology, University Hospital, Würzburg, Germany). BS153 cells were obtained from Dr. Alexander Schulte (Department of Neurosurgery, University Medical Center Hamburg-Eppendorf, Hamburg, Germany) and originally generated by Dr. Adrian Merlo (Jones et al., 2001). All GBM cells were cultured in DMEM medium supplemented with 10% FBS and 1% penicillin/streptomycin (Invitrogen). All cell lines were incubated in a humidified 5% CO_2_ atmosphere at 37°C and passaged twice weekly.

### Cytotoxicity Assays

The resazurin (Promega, Mannheim, Germany) reduction assay was performed to assess cytotoxicity of neoambrosin (**1**) and damsin (**2**) in a concentration range of 0.001–1000 μM as previously described (Kuete and Efferth, 2013). The IC_50_ values have been calculated from dose response curves and resistance ratios were determined by dividing the IC_50_ of resistant cells by the IC_50_ of the corresponding parental cells. Each assay has been done thrice with six replicates for each concentration.

### Epidermal Growth Factor Receptor Signaling Proteins Analyses by SDS-PAGE and Immunoblotting

Tumor cells (500,000 cells/well) were treated with neoambrosin (**1**) and damsin (**2**), harvested after 24 h, and washed with PBS. Total proteins were extracted by the Mammalian Protein Extraction Reagent (M-PER) (Thermo Scientific, Rockford, IL, USA) containing protease inhibitor and phosphatase inhibitor (Roche, Mannheim, Germany). Cells were shaken at 4°C for 30 min, cell lysates were centrifuged at 14,000 ×*g* for 15 min and supernatants were transferred to new tubes. Protein concentrations in lysates were detected by NanoDrop1000 (PEQLAB, Erlangen, Germany). Thirty micrograms of protein were electrophoresed on 10% SDS-polyacrylamide gels and blotted onto polyvinylidene difluoride (Ruti^®^-PVDF) membrane (Millipore Corporation, Billerica, MA, USA) using wet sandwich blotting. Membranes were washed using Tris-buffered saline containing 0.5% Tween-20 (TBST), then blocked with 5% (w/v) bovine serum albumin in TBST for 1 h at room temperature. Membranes were incubated at 4°C overnight with primary antibodies (Cell Signaling Technology, Frankfurt, Germany) including: phosphorylated EGFR (Tyr1068) (1:1000), phosphorylated STAT5 (Tyr694) (1:1000), phosphorylated Akt (Ser473) (1:1000), phosphorylated MAPK (Thr202/Tyr204) (1:1000), phosphorylated Src (Tyr416) (1:1000), Src (1:1000), and β-actin (1:2000). After washing membranes three times with TBST, the blots were probed with horseradish peroxidase-linked anti-rabbit IgG secondary antibodies (1:2000) for 2 h at room temperature. Finally, LuminataTM Classico Western HRP substrate (Merck Millipore, Schwalbach, Germany) was added for 5 min in the dark. Alpha Innotech FluorChem Q system (Biozym, Oldendorf, Germany) was used for documentation and band analysis (Zhao et al., 2015).

### Molecular Docking

Molecular docking predicts *in silico* the interaction, affinity, and geometry of ligands with target proteins. The affinity of neoambrosin (**1**) and damsin (**2**) to c-Src kinase domain compared with the known c-Src inhibitor dasatinib **(3)** (Buettner et al., 2008; Ceppi et al., 2009) was studied using Autodock4, Lamarckian algorithm (Morris et al., 2009). The compounds were prepared as 3D-structures and converted to PDBQT format. The c-Src kinase domain was obtained from the Protein Data Bank (PDB ID: 3QLG). Non-standard residues of protein (HETATM) were excluded, hydrogens added, non-polar hydrogen atoms merged, missing atoms supplemented and attached to solvation parameters. Afterward, a grid box was assigned to cover the c-Src kinase domain (PDBQT format). Docking parameters were set to 250 runs and 2,500,000 energy evaluations for each cycle. Docking was performed three times independently to calculate mean values and standard deviations of lowest binding energies and predicted inhibition constants (Pki), which were obtained from the docking log files (dlg). Binding poses between ligands (neoambrosin (**1**), damsin (**2**) and dasatinib **(3)**) and the macromolecule (c-Src kinase domain) were visualized by Visual Molecular Dynamics software (University of Illinois, Champaign, IL, USA).

## Results

### Identification and Structure Elucidation of Neoambrosin (**1**) and Damsin (**2**)

**FIGURE 1:**
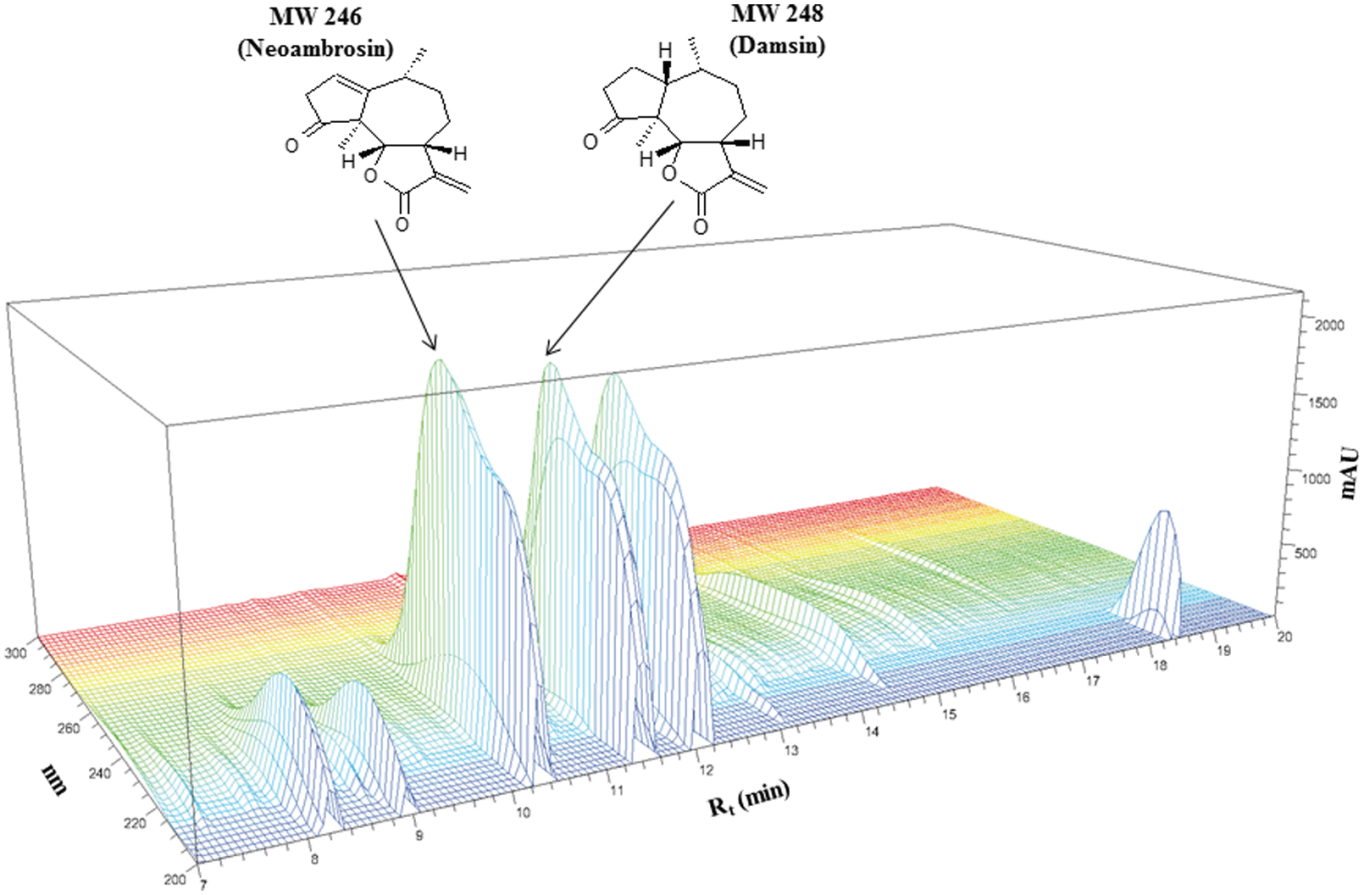
**Chemical structures of neoambrosin (**1**) and damsin (**2**) and HPLC profiling of *Ambrosia maritima* L. dichloromethane (DCM) extract**.

Two compounds **1** and **2** were identified from pure DCM extract of the whole plant of *A. maritima* L. (**Figure 1**). Neoambrosin (**1**) was obtained as colorless oil. Its structure was established by NMR. The ^13^C NMR spectrum revealed 15 signals. According to HSQC, they originated from CH_3_ groups (δ 1.14/15.2, δ 1.18/21.5), CH_2_ groups including one olefinic [δ (1.77, 1.82)/31.4, δ (2.06, 2.15)/24.7, δ (2.74, 3.22)/40.7, (δ 5.64, 6.19)/120.5], CH groups including one olefinic (δ 2.93/40.2, δ 3.49/44.8, δ 4.55/81.6, δ 6.03/125.7), and quaternary carbons (δ 59.9, 140.8, 150.4, 172.2, 216.2). These spectra data were suggestive of SLs. The skeleton and the relative configuration of the pseudoguaianolide (**1**) was confirmed from 2D NMR analysis (including COSY, HMBC, and NOESY) and comparison with literature values from previous studies. The 1D and 2D spectral data of this compound completely matched those of neombrosin (**1**), previously isolated from the aerial parts of *A. maritima* (Salam et al., 1984) and the vegetative parts of a related species, *A. hispida* by Herz et al. (1981).

Damsin (**2**) was isolated as colorless oil. Its ^13^C NMR spectra also contained 15 signals. The lack of two olefinic resonances suggested **2** as a reduced form of **1**. Based on HSQC correlations, two CH_3_ groups (δ 1.08/14.0, δ 1.08/16.0), five CH_2_ groups including one olefinic exo-methylene (δ 1.75,1.80/33.6, δ 1.80, 2.01/24.1, δ 1.80, 2.01/25.9, δ 2.24, 2.48/36.2, δ5.54, 6.28/121.1), four CH groups (δ 2.20/34.5, δ 2.01/46.3, δ 3.30/44.6, δ 4.53/81.9) and four quaternary carbons (δ 55.1, 139.7, 170.3, 219.1) were identified. The HMBC correlations indicated another pseudoguaianolide. The relative stereochemistry and the structure matched that of damsin, as previously reported by Herz et al. (1981).

### Cytotoxicity of Neoambrosin (**1**) and Damsin (**2)**

**FIGURE 2:**
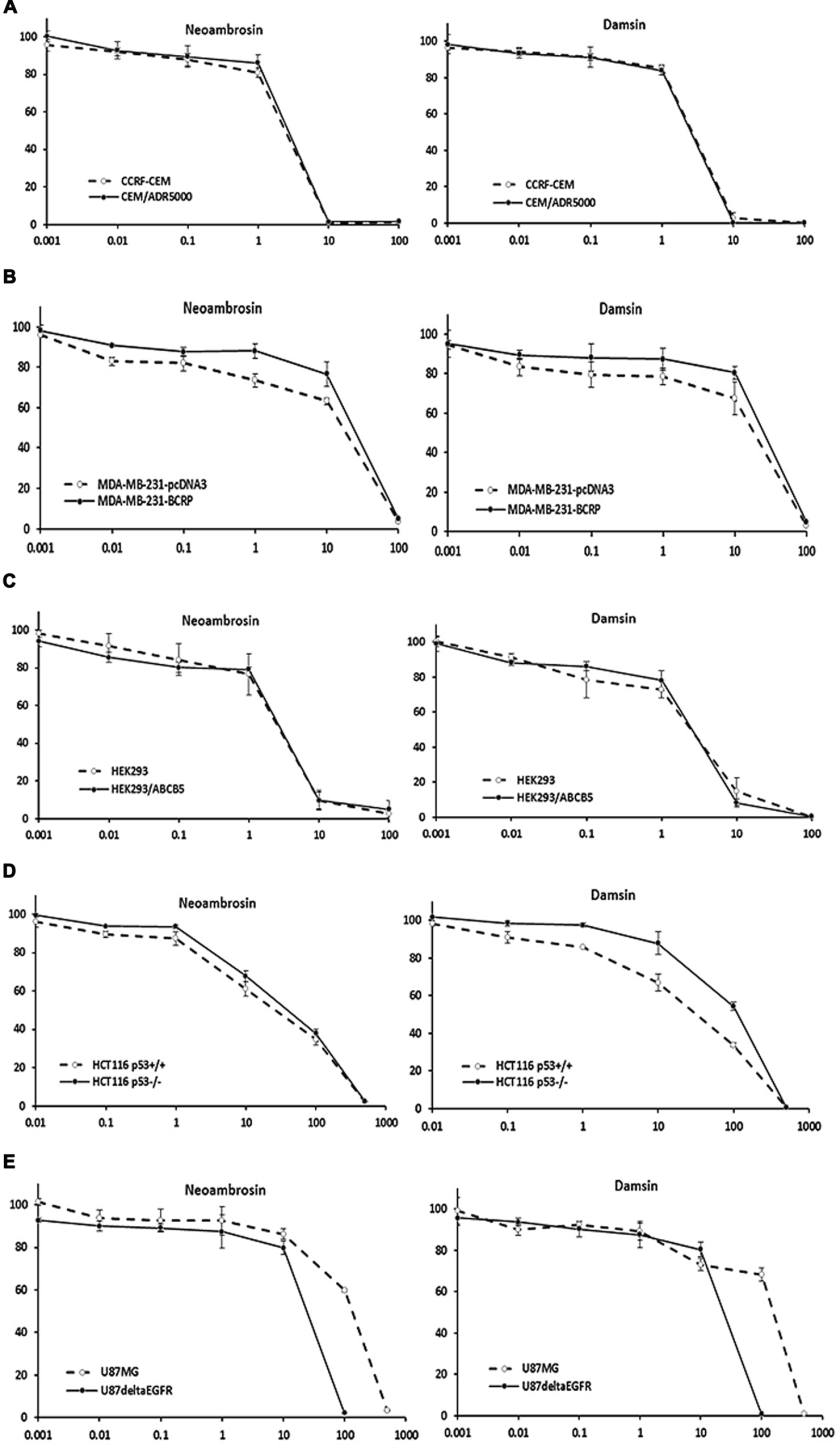
**Dose response curves of neoambrosin (**1**) and damsin (**2**) as determined by resazurin assays.** **(A)** Cytotoxicity against drug-sensitive parental CCRF-CEM tumor cells and their *P*-glycoprotein (*P*-gp; *MDR1/ABCB1*)-expressing, multidrug-resistant subline, CEM/ADR5000. **(B)** Cytotoxicity against MDA-MB-231-pc DNA cells and their BCRP-transduced subline, MDA-MB-231-BCRP. **(C)** Cytotoxicity against HEK293 cells and their *ABCB5*-transfectant subline, HEK293/ABCB5. **(D)** Cytotoxicity against HCT116 cells (p53^+/+^) and their knockout subline (p53^-/-^). **(E)** Cytotoxicity against U87MG cells and their EGFR- transduced subline, U87MGΔEGFR. The dose response curves show mean values ± SD of three independent experiments with each six parallel measurements.

Neoambrosin (**1**) and damsin (**2**) were tested in a range from 10^-3^ to 10^3^ μM in resazurin assays. Firstly, the effects of **1** and **2** on drug sensitive CCRF-CEM and resistant CEM/ADR5000 cells were analyzed. No cross-resistance was observed (**Figure 2A**; **Table 1**). The IC_50_ values were similar in both cell lines (degrees of resistance were 1.07 and 1 for **1** and **2**, respectively). Similarly, BCRP-transfected MDA-MB-231 cells were not cross-resistant to these compounds (**Figure 2B**; **Table 1**). Multidrug-resistant *ABCB5*-transfectants did also not show resistance toward these SLs (**Figure 2C**; **Table 1**).

**Table 1:** IC_50_ values of different drug-sensitive and -resistant cell lines of neoambrosin (**1**) and damsin (**2**).

Compound	Cell lines		Resistance ratio (RR)
	**CCRF-CEM**	**CEM/ADR5000**	
Neoambrosin	4.5 ± 0.2 μM	4.8 ± 0.2 μM	1.07
Damsin	4.8 ± 0.3 μM	4.7 ± 0.2 μM	1
	**MDA-MB-231-pcDNA3**	**MDA-MB-231-BCRP**	
Neoambrosin	29.9 ± 1.7 μM	43.3 ± 4.9 μM	1.4
Damsin	33.8 ± 8.4 μM	46.4 ± 2.6 μM	1.4
	**HEK293**	**HEK293/ABCB5**	
Neoambrosin	4.5 ± 0.6 μM	4.8 ± 0.2 μM	1.07
Damsin	4.5 ± 0.1 μM	4.6 ± 0.6 μM	1
	**HCT116 P53^+/+^**	**HCT116 P53^-/-^**	
Neoambrosin	26.5 ± 6.9 μM	39.4 ± 6.4 μM	1.5
Damsin	32.5 ± 6 μM	133.6 ± 18.4 μM	4.1
	**U87MG**	**U87ΔEGFR**	
Neoambrosin	132.2 ± 2.4 μM	24.1 ± 1.4 μM	0.18
Damsin	154.4 ± 8.9 μM	24.1 ± 1.5 μM	0.15


Then, neoambrosin (**1**) and damsin (**2**) were tested on wild-type HCT116 p53^+/+^ and HCT116 p53^-/-^ knockout cells (**Figure 2D**; **Table 1**). Both cell lines showed sensitivity to neoambrosin (**1**) (degree of resistance was 1.5), whereas knockout cells exhibited 4.1-fold cross-resistance to damsin (**2**) compared to wild-type cells.

Surprisingly, U87.MGΔEGFR cells were hypersensitive toward both compounds as compared to U87MG wild-type cells. The degrees of resistances were 0.18 and 0.15 for neoambrosin (**1**) and damsin (**2**), respectively (**Figure 2E**; **Table 1**). This phenomenon is known as collateral sensitivity (CS) (Hall et al., 2009) and suggests that both SLs may target proteins involved in EGFR signaling.

### Inhibition of EGFR Signaling Cascades by Neoambrosin (**1**) and Damsin (**2**)

**FIGURE 3:**
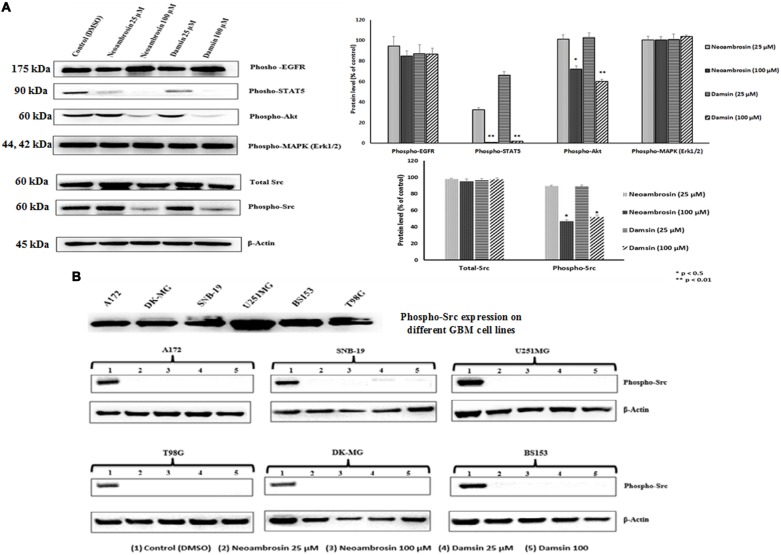
**Western blot analysis of the effect of neoambrosin (**1**) and damsin (**2**) on EGFR and its downstream signaling cascade proteins.** **(A)** Effect on phosphorylation of ΔEGFR, STAT5, Akt, MAPK (Erk1/2), c-Src, and total c-Src. Bands were normalized to β-actin in order to obtain numerical values (Mean ± SEM). Bars of the average and error bars of three independent experiments are shown. Statistical analysis was done by paired student’s *t*-test ^∗∗^*p* < 0.01,*^∗^p* < 0.05; paired *t-*test. **(B)** Effect on phosphorylation of c-Src in different GBM cell lines that highly overexpressed c-Src (A172, DK-MG, SNB-19, U251.MG, BS153, and T98G). β-actin was used as loading control.

In order to have a closer insight into the phenomenon of CS of U87.MG cells transfected with deletion-activated EGFR toward neoambrosin (**1**) and damsin (**2**), the phosphorylation status of EGFR signaling proteins as parameter of their activation during signal transduction was investigated. Two concentrations of each compound were selected: 25 μM (∼IC_50_) and 100 μM (4 × IC_50_). Both compounds inhibited STAT5 and AKT phosphorylation at 100 μM, whereas other signaling proteins were not affected, including EGFR (**Figure 3A**). It was surmised that STAT5 and AKT activation works independently from EGFR activation (Okutani et al., 2001; Haynes et al., 2003; Summy and Gallick, 2006). Then the two compounds **1** and **2** were hypothesized to target c-Src. Remarkably, they inhibited c-Src autophosphorylation at Tyr416, whereas non-phosphorylated (total) c-Src was not affected. These results indicated that both compounds targeted c-Src and silenced its downstream signaling.

For confirmation, six other glioblastoma cell lines with known c-Src overexpression were investigated. Indeed, neoambrosin (**1**) and damsin (**2**) inhibited c-Src phosphorylation in all concentrations tested (**Figure 3B**).

### *In silico* Binding of Neoambrosin and Damsin to c-Src

**FIGURE 4:**
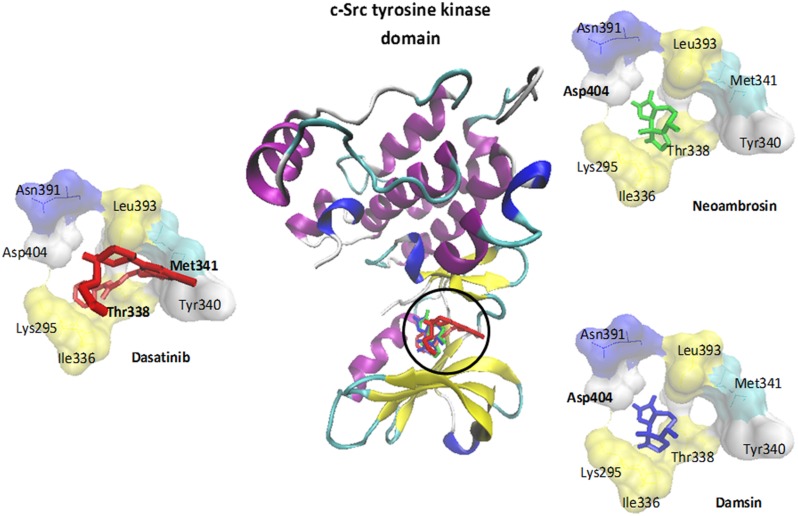
**Molecular docking of dasatinib (**3**), neoambrosin (**1**), and damsin (**2**) to the c-Src kinase domain.** The protein was represented by its secondary structure in a new carton format, whereas ligands were represented in dynamic bond format with different colors: dasatinib (**3**) (red), neoambrosin (**1**) (green) and damsin (**2**) (blue). Amino acids (AA) involved in H-bonds were shown in a bold font Met341 and Thr338 (dasatinib); Asp404 (neoambrosin and damsin).

*In silico* molecular docking of the c-Src kinase domain to both SLs and dasatinib (**3**) as positive control, were performed. The binding energy of dasatinib (**3**) to c-Src was slightly lower than those of neoambrosin (**1**) and damsin (**2**) (**Table 2**). Interestingly, all three compounds bound to the same site on the c-Src kinase domain. The c-Src kinase domain consists of characteristic small amino-terminal and large carboxyl-terminal lobes. The catalytic ATP binding site lies in a cleft between the two lobes. These lobes move relative to each other and open or close the cleft. The open form is necessary to allow access of ATP to phosphorylate c-Src. The residues, which are essential for c-Src phosphorylation are Lys295 and Asp404. Any molecule able to interrupt the open/closed c-Src form is considered to have an inhibitory effect (Hanks et al., 1988; Roskoski, 2004). Importantly, neoambrosin (**1**), damsin (**2**), and dasatinib (**3**) have the ability to bind to the cleft between the lobes. Although dasatinib revealed better affinity, both SLs bind nevertheless to the same binding site as dasatinib (**3**) (**Table 2**; **Figure 4**). This result explains the inhibition of c-Src phosphorylation by neoambrosin (**1**) and damsin (**2**).

**Table 2:** Molecular docking of dasatinib (**3**), neoambrosin (**1**), and damsin (**2**) to the c-Src kinase domain.

Compound	Lowest binding energy (kcal/mol)	Pki (μM)	AA involved in H-bonds
Dasatinib	–9.85 ± 0.03	0.06 , <0.00	Thr338, Met341
Neoambrosin	–7.23 ± <0.00	4.99 , <0.00	Asp404
Damsin	–7.25 ± 0.01	4.8 , 0.01	Asp404


*Shown are lowest binding energy, predicted inhibition constant (Pki), and amino acids (AA) involved in hydrogen bonding. Each docking experiment has been repeated three times.*

## Discussion

Sesquiterpene lactones gathered attention for cancer treatment (Lopez-Anton et al., 2007; Staudt, 2010). The cytotoxicity of pseudoguaianolide SLs, neoambrosin (**1**) and damsin (**2**) from *A. maritima* L. was tested. Neoambrosin (**1**) and damsin (**2**) inhibited all cell lines tested in the micromolar ranges. While the cytotoxicity of damsin (**2**) against cancer cells has been reported elsewhere (Villagomez et al., 2013), neoambrosin’s cytotoxic activity is reported here for the first time to the best of our knowledge.

ABC-transporter-mediated MDR leads to reduced chemotherapeutic drugs accumulation inside the cells, including anthracyclines, *Vinca* alkaloids, colchicine, epipodophyllotoxins, taxanes, camptothecins, imatinib, methotrexate, and mitoxantrone (Raaijmakers et al., 2006; Efferth et al., 2008; Fletcher et al., 2010). Among the 48 members of the ABC transporter family, *P*-gp and BCRP have been extensively characterized (Gros et al., 1986; Ueda et al., 1986; Doyle et al., 1998). Frank et al. cloned a novel ABC transporter named ABCB5, which controls progenitor cell fusion by altering the membrane potential in normal melanocytes expressing the stem cell marker CD133 (Frank et al., 2003). ABCB5 is closely related to *P*-gp and confers doxorubicin resistance in malignant melanoma (Frank et al., 2005). Furthermore, it mediates resistance to paclitaxel and docetaxel (Kawanobe et al., 2012). Cell models, which overexpressed these three ABC transporters were used. Importantly, they did not confer cross-resistance to neoambrosin (**1**) and damsin (**2**), indicating that they are not substrates for any of these three transporters. This implies that these SLs may be useful to treat MDR- tumors.

One of the most frequently mutated genes is the *TP53.* It is impaired in approximately 50% of all tumors (Nigro et al., 1989; Olivier et al., 2002). As transcription factor, p53 maintains the genomic integrity after genotoxic stress by inducing cell cycle arrest, DNA repair, and apoptosis (Vogelstein et al., 2000). Activated p53 spurs p21/WAF1, an inhibitor for G2/M-specific cell division control protein 2 kinase and cyclin-dependent G1 kinase subsequently leading to G2/M and G1 checkpoint control. Failure in arresting cells at both checkpoints due to mutated p53 confers drug resistance (Agarwal et al., 1995; Piovesan et al., 1998). HCT116 p53^-/-^ cells, which have been reported for their resistance to 5-FU, irinotecan, and oxaliplatin (Boyer et al., 2004) were tested. Interestingly, wild-type and knockout HCT116 cells were sensitive to neoambrosin (**1**), while knockout cells were cross-resistant to damsin (**2**). This indicates that both SLs may activate wild-type p53 triggering their programmed death. This result is in agreement with a previous study on another SL, parthenolide, which activated p53 by enhancing ubiquitylation and degradation of the p53-negative regulator Mdm2 (Gopal et al., 2009). The sensitivity of p53-knockout cells to neoambrosin (**1**) could be due to targeting other molecular determinants, whereas damsin (**2**) failed to address these targets. This result warrants more detailed investigation in the future.

Epidermal growth factor receptor and its downstream signaling proteins are associated with MDR and up-regulated co-expression of ABC-transporters (Hirsch-Ernst et al., 1995; Garcia et al., 2006; Katayama et al., 2007; Hoffmann et al., 2011). Oncogenic ΔEFGR confers resistance by inducing expression of the anti-apoptotic protein Bcl-xL (Nagane et al., 1998). Several *EGFR* mutations have been described. A common mutant is ΔEFGR, which causes poor prognosis and survival times, especially in glioblastoma patients (Shinojima et al., 2003). An 801 bp in-frame deletion of the extracellular EGFR domain results in ligand-independent receptor activation, consequent auto-phosphorylation of tyrosine residues in the kinase domain and induction of downstream signaling (Nishikawa et al., 1994). Surprisingly, neoambrosin (**1**) and damsin (**2**) revealed CS in U87MGΔEGFR cells compared to U87MG wild-type cells. CS represents a strategy to preferentially kill resistant cells (Hall et al., 2009). CS is well known from MDR phenotypes with *P*-gp overexpression. Several hypotheses attempted to explain CS, e.g. increased production of reactive oxygen species (ROS) in expense of ATP hydrolysis, expelling endogenous vital substrates, exploitation of energetic sensitivities and plasma membrane disruption (Pluchino et al., 2012). CS in EGFR-expressing cells has been recently described for the first time by us (Saeed et al., 2014). SLs induce oxidative stress and disturb the redox status by increased ROS production through interaction with the sulfhydryl moiety in the cysteine of the antioxidant glutathione (Sun et al., 2010). This is in accordance with the assumption that CS occurs because of ROS accumulation. However, this explanation may be insufficient, because in this case all other resistant cell lines tested by us should also exert CS because of glutathione abundance in all these cell lines.

**FIGURE 5:**
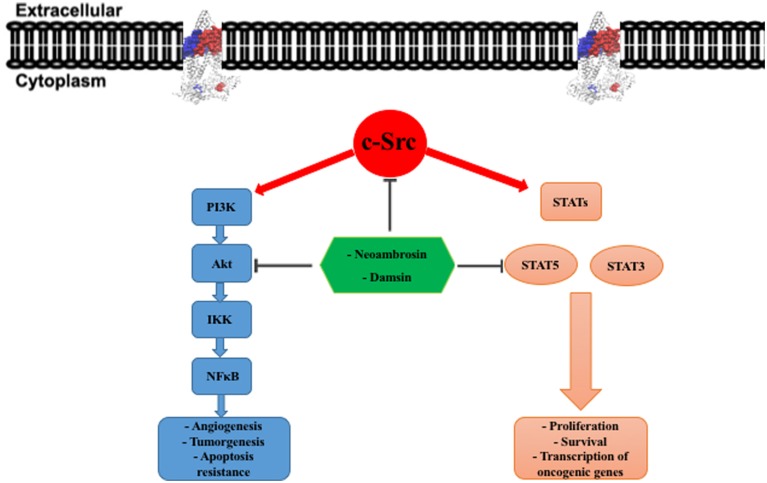
**c-Src downstream signaling pathways.** In tumor cells, overexpression of c-Src leads to constitutive activation of PI3K/Akt and STATs (STAT3 and STAT5) pathways. This activation governing cancer development and progression. PI3K, phosphatidylinositol-3-kinase; IKK, IκB kinase; NFκB, nuclear factor kappa-light-chain-enhancer of activated B cells; STAT, signal transducers and activators of transcription.

To further explore CS, we performed phosphorylation-specific immunoblotting analyses of EGFR and its downstream signal transducers. Interestingly, only AKT and STAT5 phosphorylation was downregulated, whereas EGFR and MAPK remained unaffected by the two compounds. This result raises the question about other targets of these SLs. Another key player of EGFR signaling is the oncogenic tyrosine kinase, c-Src. Worth to mention, inhibition of c-Src kinase activity diminished tyrosine phosphorylation of STAT5 in K562 cells, indicating that Src activates STAT5 (Okutani et al., 2001; Silva, 2004; **Figure 5**). Furthermore, c-Src activated AKT through the formation of a complex with regulatory subunit p85 of PI3K with subsequent activation of the PI3K/Akt pathway (Haynes et al., 2003; Summy and Gallick, 2006; **Figure 5**). Thus, c-Src inhibition could silence STAT5 and AKT phosphorylation.

Src was discovered in Rous sarcoma virus that causes transmissible tumors in chicken (Rous, 1911; Martin, 1970; Courtneidge, 2002). The viral gene product, *v-Src*, is a tyrosine kinase with a cellular homolog, c-Src (Czernilofsky et al., 1980). Upon c-Src activation, it becomes an oncogene involved in adhesion, growth, angiogenesis, and progression (Irby and Yeatman, 2000). Several studies reported elevated expression and kinase activity of c-Src in many tumor cells as compared with normal cells, suggesting that c-Src is crucial for cancer development and progression (Cartwright et al., 1989; Budde et al., 1994; Muthuswamy et al., 1994; Irby and Yeatman, 2000). c-Src has a great metastatic potential (Yeatman, 2004). The activity of c-Src tyrosine kinase interacts with receptor of tyrosine kinases such as EGFR in promoting tumor growth. The combination of EGFR and c-Src expressions in fibroblasts leads to synergistic tumorigenicity, if compared with EGFR or c-Src alone (Maa et al., 1995; Mao et al., 1997; Tice et al., 1999; Irby and Yeatman, 2000). c-Src activation increased growth rates and invasion characteristics of tumors. It also decreased apoptosis in tumor cells (Irby and Yeatman, 2000). Therefore, it is generally assumed that inhibition of c-Src function represents an attractive target to stop tumor growth. The recent use of c-Src inhibitors or antisense therapy in nude mouse studies, pancreatic cell growth, and leukemia cells supports the validity of this concept (Staley et al., 1997; Lutz et al., 1998; Roginskaya et al., 1999).

It has been postulated that c-Src could be a target for neoambrosin (**1**) and damsin (**2**) and that CS emerged in EGFR-expressing cells due to c-Src inhibition. We further investigated, how c-Src expression is affected in different other brain tumor models with c-Src overexpression upon treatment with these two SLs. Interestingly, both compounds inhibited c-Src phosphorylation not only in ΔEGFR-transfected cells, but also in six other c-Src-overexpressing cell lines. Further confirmation came from *in silico* molecular docking studies. Neoambrosin (**1**), damsin (**2**), and dasatinib (**3**) bound to the cleft between the two c-Src’s lobes. These small amino-terminal and large carboxyl lobes are of crucial importance for ATP binding to activate c-Src, thus, blocking of this cleft leads to impaired c-Src activation. These results indicate that neoambrosin (**1**) and damsin (**2**) are interesting c-Src inhibitors. Recent studies have shown that targeted inhibition of c-Src by dasatinib leads to decreased growth, and induced cell-cycle arrest and apoptosis in a subset of thyroid cancer and breast cancer cells. This therapeutic approach has been verified *in vitro* by immunoblotting analysis (Nautiyal et al., 2009; Chan et al., 2012). In this respect, it is remarkable that neoambrosin (**1**) and damsin (**2**) reveal similar c-Src-inhibiting features as dasatinib. Hence, it is reasonable to conclude that the inhibition of c-Src function (i.e., phosphorylation) led to the inhibition of tumor cell growth, which we observed in resazurin assays.

A previous study demonstrated that damsin (**2**) inhibited STAT3 and NF-κB expression in Jurkat and Hela cells by interfering with their signaling pathways (Villagomez et al., 2013). This result is in accordance with our finding that damsin (**2**) inhibited a master regulator upstream of STAT3 and NF-κB. Interestingly, c-Src directly activates STAT3, and NF-κB is activated by the PI3K/Akt pathway (Silva, 2004; Summy and Gallick, 2006). AKT enhances the transcriptional activity of NF-κB by phosphorylation and subsequent degradation of inhibitor of κB (IκB) (Bai et al., 2009). Moreover, several studies stated that PI3K/Akt pathway regulates the expression of *P*-gp (ABCB1) and BCRP (ABCG2) (**Figure 5**) (Barancík et al., 2006; Chiarini et al., 2008; Pick and Wiese, 2012).

Noteworthy, there is a cross talk between c-Src and p53. c-Src suppressed the apoptotic p53 pathway (Courtneidge, 2002; Polonio-Vallon et al., 2014). This may explain the sensitivity of wild-type 53 cells to neoambrosin (**1**) and damsin (**2**) and that they probably exert their cytotoxicity toward wild-type 53 cells by c-Src inhibition.

## Conclusion

Neoambrosin (**1**) and damsin (**2**) isolated from the whole plant of *A. maritima* L. possess remarkable cytotoxicity against different drug-resistant tumor cell lines. Both SLs silenced c-Src phosphorylation, which represents an interesting approach for argeted therapy, because c-Src is activated in many human cancers (Yeatman, 2004). The remarkable CS of neoambrosin (**1**) and damsin (**2**) toward EGFR-expressing cells opened prospects for synergistic tumor killing if combined with established anticancer drugs. Clinically, tumors develop MDR and activate alternative pathways to survive and disseminate. Thus, combination therapies with these two SLs might improve cure rates of cancer patients (Shapiro, 1955; Devita et al., 1970; Rougier and Lepère, 2005; Li et al., 2014).

## Conflict of Interest Statement

The authors declare that the research was conducted in the absence of any commercial or financial relationships that could be construed as a potential conflict of interest.

## ABBREVIATIONS

ABC: ATP-binding cassette
CS: collateral sensitivity
DCM: dichloromethane
EGFR: epidermal growth factor receptor
GBM: glioblastoma multiforme
MDR: multidrug resistance
TP53: tumor suppressor p53.
